# Editorial: Exploring complex biosphere molecular signaling networks: plant-microbes symbiosis at microscopic to macroscopic levels

**DOI:** 10.3389/fpls.2023.1199162

**Published:** 2023-05-26

**Authors:** Yutao Peng, Chung-Yu Guan, Ashok Zachariah Samuel

**Affiliations:** ^1^ School of Agriculture, Sun Yat-Sen University, Shenzhen, China; ^2^ Department of Environmental Engineering, College of Engineering, National Ilan University, Yilan, Taiwan; ^3^ Research Organization for Nano And Life Innovation, Waseda University, Tokyo, Japan

**Keywords:** Raman spectroscopy, plant microbe symbiosis, smart analytical techniques, hplc, chemical constitution of crops, plant metabolites

## Introduction

Plants have evolved to live on earth using basic chemicals, such as, CO_2_, water, and minerals in air and soil. This knowledge helped human beings develop industrial agriculture to build and maintain civilizations, to domesticate animals, and to meet the food demands of growing population. However, crop production engineering using chemical fertilizers/pesticides, overexploitation of soil resources disregarding subtle balances in the ecosystem, and climate change have created a crisis (Timmis and Ramos, 2021). Our realization that soil is a highly interconnected ecosystem with interspecies chemical exchange through, for instance, symbiosis, has opened avenues for developing sustainable agricultural practices protecting the environment (Zipfel and Oldroyd, 2017). Moreover, the quality of the agricultural products depends heavily on the biotic and abiotic environmental factors plants experience in the environment. The environmental factors that affect crops need to be assessed scientifically and documented, rather than relying on sensory perception to assess nutritional quality of the products. Further, fundamental investigations should also be conducted to better understand the symbiosis between microbes and plants from a molecular perspective. Studies in the recent past have revealed several molecular aspects of soil microbes’ entry into legume plants’ root system, subsequent nodulation of root, and effective nitrogen fixation. Discovery of nod factor (e.g., oligosaccharide derivative produced by *R. meliloti* (Truchet et al., 1991)) revealed the molecular mechanism of microbe entrapment in root hairs (Sahlman and Fahraeus, 1963). Subsequent studies revealed the role of symbiosis entry receptors (e.g., LYK3), and hence the corresponding genes, in the effective infection process. Similarly, phosphate levels in the environment and Ca^2+^ spiking are essential for causing appropriate changes in the root hair phenotype and for infection to proceed beyond the microbe entrapment in root hairs (Gilroy and Jones, 2000; Parry, 2018; Bono et al., 2020; de Bruijn, 2020a; de Bruijn, 2020b). Next generation smart analytical tools are also being developed for automated diagnosis of plants by measuring specific molecular species (e.g., volatile organic compounds (VOC), plant metabolites, etc.) (Lew et al., 2020). Accessible and affordable robots, controlled by scientific data-driven artificial intelligence modules, are being developed for specific applications in agriculture.

## Chemical analyses of plants and products

During different developmental stages, plants produce distinct chemicals (e.g., ethylene). At several instances, chemical profile changes can be correlated with pathogen infection much before real symptoms starts to appear. For instance, tomato leaves infected with three different pathogens showed accurately identifiable VOC profiles before visible symptoms appeared (Li et al., 2019). Handheld and smartphone-based spectrometers along with spectral matching applications through mathematical routines find unique applications in agricultural fields (Samuel et al., 2021). Parlamas et al. shows possible field application of Raman spectrometer for detecting *Fusarium oxysporum* f. sp. *cubense* infection in banana (*Musa* spp.) plants. Changes in carotenoid expression in banana leaves clearly distinguished infected plants (biotic stress) from healthy ones. Higgins et al. presents a comprehensive evaluation of how abiotic stresses, such as, nitrogen deficiency (ND) and drought, and biotic stresses, such as, aphid infestation and viral infections, affect common wheat. Changes in lutein, chlorophyll, pheophytin, and *beta*-carotene were estimated in leaves of those wheat plants with high-pressure liquid chromatography (HPLC). Aphid infestation was found to be the most detrimental among the stresses investigated. Raman spectral data collected directly from wheat leaves with a handheld spectrometer indicated similar reduction in carotenoid band intensity suggesting possible field applications of the technique.

Equally important is chemical composition of nutritionally and medically important molecules in plant produces. If spectral signatures corresponding to a molecule of interest is known, then a spectrometer can be calibrated (e.g., with HPLC, for instance) and predictive models (e.g., partial least square methods, machine learning modules) can be built, which will then allow subsequent quantitative chemical analysis of crops directly from spectral intensities. Ming et al. demonstrates applicability of HPLC and near IR (NIR) spectroscopy in estimating oleanolic acid (OA) and ursolic acid (UA) contents in *Chaenomelis Fructus*. Multivariate statistical and machine learning models are proposed for future real time industrial applications of the study. A comprehensive chemical analysis of parts of plants can be performed with HPLC and mass spectrometry (MS) by extracting its contents by chemical means. Anwar et al. have shown ultra-high performance liquid chromatography-mass spectrometry (UHPLC-MS) analysis of solvent extracts from roots and aerial parts of *Crotalaria burhia*, which revealed an extensive list of phytochemicals including polyphenols, saponins, flavonoids, and glycoside derivatives. Gautam et al. demonstrates changes in composition of potato tubers produced under heat stress conditions. Proteins, reducing sugars, total dry matter, and specific gravity estimates shows considerable fluctuations under heat stress. Notably, reducing sugars show drastic increase in some potato clones compared to others. Raman spectra collected from these samples showed drastic reduction in starch content, while the carotenoid content has not changed. Mg^2+^ is an important mineral that plays crucial roles in photosynthesis, carbohydrate partitioning etc. in plants (e.g., rice), which affects crop yield and quality. Zhi et al. examined genetic and molecular mechanism of Mg^2+^ uptake and translocation. Translocation of Mg^2+^from root to stem was estimated using inductively coupled plasma mass spectrometry (ICP-MS). A gene that plays a major role in the uptake and translocation of Mg^2+^ in rice populations has been proposed based on the results. Correlating genetic information and metabolic information is an important area requiring future exploratory studies.

## Summary and perspectives

The research articles published in this Research Topic provides specific examples of utility and adaptability of general analytical techniques in agricultural research and field applications. Further, a literature review by Chen et al. discusses the effective utilization of agricultural residues, such as, straw return and straw biochar, for improving soil properties. Automated field deployment of sensitive analytical laboratory techniques by integrating robotics and artificial intelligence is expected to revolutionize sustainable agricultural initiatives.

## Author contributions

All authors listed have contributed to realizing the article collection under this Research Topic. AZS wrote the editorial and all the authors approved it for publication.
